# Examining Quality-of-Life Priorities of Older Adults Receiving Hemodialysis: A Q-Methodology Study

**DOI:** 10.1016/j.xkme.2026.101348

**Published:** 2026-04-01

**Authors:** Anika Lucas, Jeanette Rutledge, John Roberts, Cathleen Colon-Emeric, Rasheeda Hall

**Affiliations:** 1 Renal Section, Department of Medicine, Durham Veterans Affairs Healthcare System, Durham, NC; 2 Geriatric Research Education and Clinical Center, Durham Veterans Affairs Healthcare System, Durham, NC; 3 Division of Nephrology, Department of Medicine, Duke University, Durham, NC; 4 Center for the Study of Aging and Human Development, Duke University, Durham, NC; 5 Division of Geriatrics, Department of Medicine, Duke University, Durham, NC

**Keywords:** Quality of life, older adults, hemodialysis

## Abstract

**Rationale & Objective:**

Older adults represent the fastest-growing demographic group initiating hemodialysis (HD) in the United States. Compared with older adults who do not receive HD, they commonly report a lower quality of life (QOL). However, their perspectives on QOL are poorly understood. The objective of this study was to identify and characterize QOL priorities of older adults receiving HD.

**Study Design:**

Cross-sectional study using Q-methodology.

**Setting & Participants:**

Participants were recruited from dialysis centers in and around Durham, North Carolina. Each participant sorted 35 QOL statements based on the level of agreement (eg, agree, disagree, or neutral).

**Analytical Approach:**

Factor analysis of the Q-sorts was performed using the PQ Method software. Factors were interpreted and described as QOL priorities. Demographic and clinical characteristics were summarized overall and based on factors.

**Results:**

29 older adults were recruited with a mean age of 76.2 ± 5.6 years, a median dialysis vintage of 3 (1-4.8) years, and 18 (62.1%) women. Ten (34%) participants screened positive for frailty questionnaire responses and 16 (55.2%) participants reported using an assisted device. Factor analysis revealed the following 2 distinct QOL prioritization profiles: (1) “Everyday Well-Being” and (2) “Safety and Security.” “Everyday Well-Being,” defined using 16 Q-sorts, represented a perspective that highly valued cognitive function (memory/thinking ability), spirituality, adequate pain control, and well-functioning dialysis access. “Safety and Security,” defined using 11 Q-sorts, represented a perspective that highly valued socioeconomic stability, including financial stability, access to reliable transportation, and safety. We observed no difference in age, dialysis vintage, or performance on cognitive, physical function, and frailty assessments between participants whose Q-sorts defined each prioritization profile.

**Limitations:**

Cross-sectional design, confinement to 1 geographical region.

**Conclusions:**

Using Q-methodology, we identified 2 dominant profiles of QOL priorities among older adults receiving HD. These findings highlight heterogeneity in what matters most to older adults receiving HD and the need for personalized, patient-centered approaches to evaluating and improving their QOL.

## Plain-Language Summary

Older adults receiving hemodialysis (HD) experience unique challenges, yet their perspectives on quality of life (QOL) are not well understood. We examined the QOL priorities of older adults receiving HD through a series of rankings and identified 2 distinct priority profiles. The first group of older adults focused on their everyday well-being, maintaining cognitive function, pain management, and functional dialysis access. The second group prioritized safety and security, including socioeconomic stability and recovery after dialysis treatments. Understanding what matters most to older adults receiving HD will facilitate individualized, patient-centered care and development of patient-centered interventions to improve the QOL of older adults.

The prevalence of end-stage kidney disease is highest among adults aged 75 years and older, surpassing all other age groups according to the 2024 United States Renal Data System.^1^ Older adults receiving hemodialysis (HD) experience unique challenges related to both aging and end-stage kidney disease. Compared with the general older adult population, older adults receiving dialysis have higher mortality rates, multimorbidity, symptom burden such as chronic pain and sleep disturbances, frailty, cognitive impairment, and functional impairment.^2^, ^3^, ^4^, ^5^, ^6^, ^7^ Older adults receiving dialysis report lower quality-of-life (QOL) scores than older adults not receiving dialysis.^7^^,^^8^ Given the rising trend in interventions targeting QOL in dialysis patients, efforts to appropriately measure QOL are needed.^9^, ^10^, ^11^

Despite numerous existing patient-reported outcome measures and QOL instruments, these instruments fail to capture important priorities of older adults. Specifically, in semistructured interviews, Hall et al^12^ found that social support and autonomy in daily activities were important for older adults receiving HD, but these factors were absent from the World Health Organization Quality of Life for Older Adults (WHOQOL-OLD) and Kidney Disease Quality of Life (KDQOL-36). The Centers for Medicare and Medicaid Services require QOL assessments on all patients receiving dialysis, but the KDQOL-36 instrument was initially validated in a relatively young cohort of dialysis patients; only 10% of the initial validation cohort was aged ≥75 years.^13^ It is possible that the lived experiences and priorities of older adults receiving dialysis were not optimally included in KDQOL-36 development. Therefore, to optimize the appropriateness of QOL assessment, there is a need for research on the older dialysis populations’ QOL priorities.

One approach to understanding subjectivity within a specified population is with Q-methodology. Q-methodology offers a rigorous mixed-methods approach to examine subjective attitudes and/or beliefs.^14^ Participants rank and sort statements (termed Q-sort) according to their viewpoints. Participants’ data are evaluated using factor analysis, which identifies groups of participants with shared viewpoints. This research methodology has been successfully applied in cohorts of older adults and younger patients with kidney disease.^15^^,^^16^ Therefore, the primary objective of this study was to use Q-methodology to identify QOL domains prioritized by older adults receiving dialysis.

## Methods

This was a cross-sectional study involving primary data collection via Q-methodology to understand the priorities on QOL of older patients receiving HD. This study was approved by the Institutional Review Board of Duke University (Pro00063355) and the Durham Veterans Affairs Healthcare System (MIRB# 01975/0001).

### Study Population

Patients eligible for the study were aged at least 65 years with at least 2 comorbid chronic conditions. Patients received dialysis within 15 miles of Duke University and had a dialysis vintage of at least 3 months. Patients on kidney transplant waiting lists were excluded to ensure a homogenous older adult cohort with respect to outlook on life and health status.^17^ Patients who were visually impaired, and/or could not speak and understand English, were also excluded.

### Approach

Q-methodology integrates qualitative and quantitative research techniques to examine the subjective attitudes, experiences, and/or beliefs of individuals methodically, uncovering viewpoints while incorporating statistical analysis through factor analysis.^14^ Q-methodology involves participants ranking a set of statements based on how strongly they agree with the statement.^15^ We developed our statement set to cover established QOL domains described in existing literature, including social, cognitive, physical, psychological, and spiritual well-being, end-of-life issues, spirituality, and physical environment.^18^^,^^19^ To capture patient perspectives directly, we generated statements from interview themes identified in our qualitative study of QOL priorities among older adults receiving HD.^12^ We refined and finalized the statement set (termed the Q-set) after independent evaluation and feedback from clinicians (1 social worker and 2 physicians) who provide specialized care for older adults receiving HD. The final Q-set included 35 statements, starting with the phrase, “It is important to me” (Table S1). Each phrase was printed on a card with a corresponding photo meant to capture that domain, to address issues of literacy.

**Figure 1:**
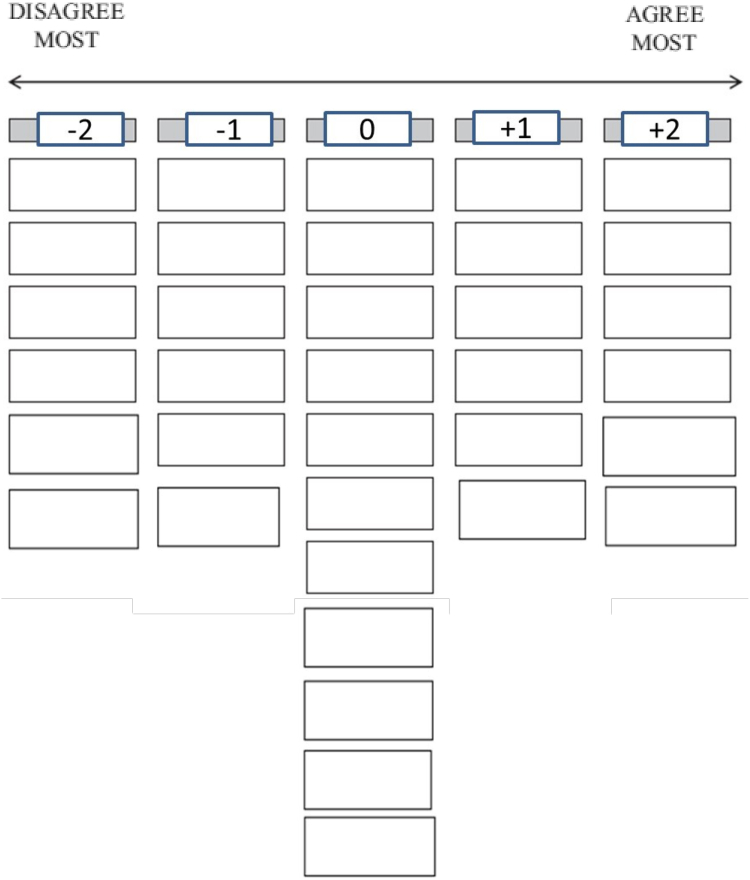
Q-sort response grid. This is a visual representation of participants’ score sheet. Participants ranked 35 predefined statements into each column ranging from “Disagree Most” to “Agree Most.”

Participants ranked all 35 statements of the Q-set (35 statements) across 5 categories (-2 to +2, strongly disagree to strongly agree), producing a Q-sort, depicted in Fig 1. A Q-sort is an individual’s complete ranking, representing a participant’s unique perspective and data point of analysis. To do the ranking, research staff presented the Q-set to participants and instructed them to sort the statement cards into the following 3 category baskets: (1) “Agree” (statements they agree with), (2) “Disagree” (statements they disagree with), and (3) “Neutral” (statements they neither disagree nor agree with). Participants were allowed to place 12 cards into each of the “Agree” and “Disagree” category baskets and 11 cards into the “Neutral” basket. Next, the participants ranked the 12 statements from the “Agree” basket by identifying the 6 statements that they agreed with most strongly (“Agree Most,” +2 in Fig 1). The remaining 6 were considered statements they agreed less with (+1 in Fig 1). Similarly, participants ranked all the statements they disagreed with from “most disagree” to “least disagree,” scored as -2 and -1, respectively. Statements placed in the “Neutral” basket were scored as 0. Finally, participants explained their selections in audio-recorded interviews with research staff for 5 minutes, highlighting the statements they strongly agreed and disagreed with.

We additionally collected data on patient demographics (age, sex, and race/ethnicity), dialysis vintage (years), and comorbid conditions (Charlson comorbidity index). We performed screenings for cognitive impairment using the Mini-Cog and frailty using the FRAIL questionnaire.^20^^,^^21^

### Data Analysis

Factor analysis was performed using the PQ Method software (Version 2.35; Schmolck). We extracted factors using centroid factor analysis and performed varimax rotation on all factors. Distinct factors were groups of Q statements with an eigenvalue of >1. Descriptive statistics were performed to describe continuous variables (mean and standard deviation) and categorial variables (counts and percentages) for each factor. Interview transcripts were reviewed for exemplary quotations.

## Results

We recruited 29 older adults receiving HD to participate in this study. The mean age of study participants was 76.1 ± 5.6 years. Additional participants’ demographic and clinical characteristics are depicted in Table 1. The Q analysis revealed 2 distinct QOL prioritization profiles or factors (eigenvalue > 1). Factor 1 was defined based on the rankings of 16 participants. Factor 2 was defined based on the rankings of 11 participants. Two participants’ data did not correspond significantly with either factor. Table 1 shows similar demographic, clinical, and functional characteristics for participants whose viewpoints were aligned with factor 1 or factor 2.

**Table 1:** Baseline Characteristics

Participant Characteristics	Mean (SD), Median (Q1-Q3), or n (%)		
	Overall N = 29	“Everyday Well-Being” (n = 16)	“Safety and Security” (n = 11)
Age, y	76.2 ± 5.6	76.1 ± 6.1	75.3 ± 4.4
Female sex	18 (62.1)	11 (68.7)	6 (54.5)
Race/ethnicity^a^			
Black race	17 (58.6)	10 (62.5)	6 (54.5)
White race	12 (41.4)	6 (37.5)	5 (45.4)
Hispanic	1 (3.4)	-	1 (9)
Time on dialysis (mo)^b^	36 (12.7-58.2)	43 (24-68)	36 (14.5-58.5)
Charlson index	7 (5.7-9)	6 (5-7.5)	8 (5-9)
Comorbid conditions^c^			
Cardiovascular disease	15 (53.6)	6 (40)	7 (63.6)
Pulmonary disease	5 (17.8)	1 (6.7)	4 (36.4)
Liver disease	1 (3.6)	-	1 (9.1)
Diabetes mellitus	12 (42.8)	5 (33.3)	5 (45.4)
Malignancy	2 (7.1)	1 (6.7)	-
Connective tissue disease	3 (10.7)	2 (13.3)	1 (9.1)
Ulcer disease	1 (3.6)	1 (6.7)	-
Abnormal cognitive screen^d^	0	0	0
Positive frailty screen^e^	10 (34.4)	6 (37.5)	3 (27.3)
Assistive device use	16 (55.2)	8 (50)	7 (63.6)

Abbreviation: SD, standard deviation.

a Represent racial/ethnic groups reported by study participants.

b Median (Q1-Q3); N = 29.

c Mini-Cog ≤ 3 indicates mild cognitive impairment or dementia.

d Comorbid condition data for n = 28.

e FRAIL Scale score of 3 or more indicates a positive frailty screen 19.

### Factor 1: Everyday Well-Being

Table 2 shows the defining statements and scores for factor 1. This perspective prioritized (ie, statements that participants agreed with the most) their independence, including maintaining their memory and ability to think (statement 6) and ability to bathe, dress, eat, and go to the toilet on their own (statement 13). In postsort interviews, participants emphasized the importance of maintaining their memory and thinking ability asserting, “I’d do anything to keep it.” Another participant stated, “If you can’t think straight, you can’t do your business.” Memory and thinking ability were perceived as essential to independence to “do things on my own,” “make decisions on my own,” and not “worry somebody else.”

**Table 2:** Defining Statements of Factor-Derived “Everyday Well-Being” Profile

Statement	Ranking Score	Z Score
6. Keep my memory and thinking ability	2	2.01
13. Am able to bathe, dress, eat and go to the toilet on my own	2	1.93
7. Have a dialysis access site that works without problems	2	1.70
2. Find strength in my faith or spiritual beliefs	2	1.58
16. Am satisfied with the care I receive from my health care team	2	1.29
8. Have my pain controlled	2	1.20
10. Don’t feel like a burden on my family and friends	1	1.01
23. Have restful sleep	0	-0.08
34. Have things I look forward to doing	-1	-0.83
19. I am satisfied with how I have lived my life	-1	-0.84

*Note*: n = 16 older adults. All statements were significant at *P* < 0.01; ranking scores range from -2 to +2 and negative scores indicate disagreement. The magnitude of Z score indicates the strength of agreement or disagreement with the statement. Z scores > 1 indicate distinguishing features or characteristics of the factor. Z scores at or near 0 indicate neutrality, whereas negative Z scores represent ideas that were least characteristic of that viewpoint (and opposed by participants).

This perspective also demonstrated strong attention to the daily impact of living with kidney failure and requiring HD. For example, functioning dialysis access (statement 7), adequate pain control (statement 8), and satisfaction with their health care team (statement 16) were highly valued. During postsort interviews, participants described, “Important to have active access… Without dialysis you die.” Regarding pain management, several participants declared, “I like to have pain control,” “nobody wants to be in pain,” and “I’m allergic to pain.” This perspective also prioritized faith and/or spiritual beliefs, which provide “the basis of living” and “the strength to keep going.” It also offered hope for participants who stated that faith and/or spiritual beliefs “make things better,” “gets me through a lot of stuff,” and are “very important.” Faith and/or spiritual beliefs included but were not limited to prayer and church attendance.

### Factor 2: Safety and Security

Table 3 shows the defining statements and scores for factor 2. This perspective prioritized fulfillment of their socioeconomic needs, including reliable transportation (statement 33), having enough money to meet their needs (statement 27), and safety at home and in their neighborhood (statement 3). During postsort interviews, participants who shared this viewpoint asserted, “If you ain’t got enough money to meet your needs, you’re in trouble” and “you need money to survive.” This perspective prioritized statements about the importance of their ability to perform more complex tasks independently such as shopping, housework, managing money, and taking medications (statement 12). One participant stated, “I still want to go shopping and pick the little things I want,” and another reported, “I manage my own bank accounts and bills.” Similarly, dialysis recovery (statement 26) also had high importance.

**Table 3:** Defining Statements for Factor-Derived “Safety and Security” Profile

Statement	Ranking Score	Z Score
33. Have reliable transportation to and from dialysis	2	2.08
27. Have enough money to meet my needs	2	1.67
26. Recover quickly after each dialysis	2	1.08
3. Feel safe in my home and neighborhood	2	1.28
12. Am able to take care of shopping, housework, managing money	1	1.05

*Note*: n = 11 older adults. *P* values were only recorded for factor 1. Ranking scores range from -2 to +2 and negative scores indicate disagreement. The magnitude of Z score indicates the strength of agreement or disagreement with the statement. Z scores > 1 indicate distinguishing features or characteristics of the factor. Z scores at or near 0 indicate neutrality, whereas negative Z scores represent ideas that were least characteristic of that viewpoint (and opposed by participants).

### Consensus QOL Statements

We observed consensus between these 2 factors regarding 15 QOL statements (Table 4). In both factors, the following statements were considered relatively important: (1) *support from family and friends* (statement 1), (2) *moving around home independently* (statement 11), and (3) *not feeling sick all the time* (statement 15). In contrast, the following statements were ranked as not important: (1) *satisfaction with the dialysis units’ appearance* (statement 4), (2) *setting a daily schedule* (statement 14), (3) *active social life* (statement 17), (4) *making medical decisions* (statement 21), (5) *an active sex life* (statement 29), (6) *traveling* (statement 30), and (7) *being in good spirits* (statement 20). Statements that were neutral in both factors were getting along with family and friends (statement 25) and talking with loved ones about how I want to live my last days (statement 28). Similarly, someone to talk to about problems (statement 31) and not being troubled by fear or worry (statement 35) were ranked either neutral or not important.

**Table 4:** Statements With Similar Rankings in Both “Everyday Well-Being” and “Safety and Security” Profiles

Statement	“Everyday Well-Being”		“Safety and Security”		Priority Level
	Ranking Score	Z Score	Ranking Score	Z Score	
1. Have support from family and friends	1	0.73	1	0.84	Important
11. Move around home independent	1	0.81	1	0.76	Important
15. Don’t feel sick all the time	1	0.68	1	0.43	Important
25. I get along with family and friends	0	-0.25	0	-0.24	Neutral
28. Talk to my loved ones about my last days	0	-0.47	0	-0.09	Neutral
4. Satisfied by HD appearance	-2	-0.96	-2	-1.14	Not important
14. I can’t set my daily schedule	-2	-1.34	-2	-1.13	Not important
17. Active social life	-2	-1.73	-2	-1.60	Not important
29. Active sex life	-2	-1.77	-2	-2.10	Not important
30. I can travel when I want to	-2	-0.99	-2	-1.27	Not important

*Note*: All listed statements are nonsignificant at *P* > 0.01 and *P* > 0.05. Ranking scores range from -2 to +2, with negative scores indicating disagreement.

Abbreviation: HD, hemodialysis.

### Participant Interviews

Postsort interviews provided explanations for participants’ rankings of Q statements (Table 5). Participants defined based on “Everyday Well-Being” emphasized their spirituality as contributing to their “strength” and critical need for dialysis access and pain control. Participants defined based on “Safety and Security” explained that they desired to not “depend on someone else.” An underlying theme of independence was shared by both QOL profiles.

**Table 5:** Representative Quotes From Older Adults Receiving Dialysis on Defining Statements for “Everyday Well-Being” and “Security” Profiles

Defining Statement		Illustrative Quotation
**“Everyday Well-Being”**		
2.	Find strength in my faith or spirituality beliefs	“If you do not have your faith and your spirit in the Lord, you need to look. And that’s where my strength comes from is that I have my faith and my spiritual beliefs.”
6.	Keep my memory and thinking ability	“It’s very important because that’s everyday life, and if you can’t think straight, you can’t handle your money, you can’t do your business. If you don’t have no memory, it’s very difficult.”
7.	Have a dialysis access site that works without problems	“Yes, its very important to have an active access because otherwise you can’t get dialysis. And without dialysis, you die.”
8.	Pain control	“As I told you before, I’m allergic to pain. I do not like a pain. Uncontrolled pain—that’s death to me.”
13.	Ability to bathe, dress, eat and go to the toilet	“It’s important to be able to go the bathroom without someone standing there watching.”
**“Safety and Security”**		
3.	Feel safe in my neighborhood	“Because I want to feel safe in my home, and in my neighborhood also. Because I don’t wanna live somewhere I’m afraid to go out the door, or afraid somebody’s gonna come in on me.”
12.	Ability to take care of shopping, housework, managing money, and taking medications on my own	“That something we all want to do by ourselves, right there. Take your own medicine, manage your own money. Housework, probably not, give that up a long time ago. Shopping—I still want to go shopping and pick up the little things that I want.”
26.	Recover quickly after dialysis	“If I don’t recover after each dialysis, it means I’m dead! And I really wouldn’t want that. So that’s why I chose that one.”
27.	Have enough money to meet my needs	“It is very important because I don’t want to have to depend on someone else to meet my needs for me.”
33.	Have reliable transportation to and from dialysis	“If I don’t have transportation to dialysis—to and from dialysis—it could mean my life, if I don’t have any way to get there. So that’s why I chose this one.”

## Discussion

Our application of Q-methodology revealed the following 2 distinct factors corresponding to 2 QOL prioritization profiles in older adults receiving HD: (1) “Everyday Well-Being” (factor 1) and (2) “Safey and Security” (factor 2). Given similarities in demographic and clinical characteristics among older adults whose rankings aligned with each profile, our findings suggest that even among participants with similar comorbid conditions, physical function, and cognitive abilities, there is heterogeneity in QOL priorities. Moreover, it highlights the critical need to assess this wide range of QOL priorities in the older dialysis population.

Our findings complement prior studies involving older adults receiving HD. Factor 1 (“Everyday Well-Being”) was highly focused on the daily experience of living with kidney disease, valuing adequate pain control and physical function, consistent with qualitative and quantitative studies on QOL among older adults receiving HD.^12^^,^^22^^,^^23^ In addition to those studies, our findings demonstrate that socioeconomic stability is another key QOL priority. It is well established that low socioeconomic status is associated with incident end-stage kidney disease among older adults^24^, ^25^, ^26^ and emerged as an important theme in focus groups of older adults receiving HD in a study aimed at improving physical function and social support.^24^ Specifically, the need for transportation and maintaining employment were highlighted in discussions with older adults.^24^

Although we observed varied QOL priorities, there were similarities across the profiles in rankings of statements related to independence and end-of-life discussions. First, both profiles prioritized (ranked “Agree”) maintaining independence. Our results are consistent with those of Ramer et al^27^ who previously reported that maintaining independence was the highest ranked priority of adults aged 60 years or older with advanced chronic kidney disease (chronic kidney disease G4/5). Second, both profiles deprioritized (ranked “Neutral”) end-of-life discussions with loved ones. Although this may be unexpected given the high mortality rate in kidney failure, other studies have shown similar findings. Logan et al^28^ found that older adults with kidney disease deprioritized advanced care planning relative to physical function and physical health. Russwurm et al^29^ also observed that <50% of older adults receiving dialysis contemplated their end-of-life wishes. Although a focus on end-of-life discussions may not be common among older adults receiving HD, it remains highly relevant for clinicians to offer advance care planning discussions to facilitate other QOL priorities that may be more important to their patients (eg, faith and social support).^30^, ^31^, ^32^

Our study findings reveal further limitations of existing QOL instruments. Although spirituality and independence are incorporated in the WHOQOL-OLD instrument and independence is also assessed within KDQOL-36, neither instrument may be sufficient to uncover socioeconomic stability. Conversely, domains included in KDQOL-36 and WHOQOL-OLD on sexual intimacy and mood were deprioritized by participants, suggesting a misalignment between patient values and existing QOL measures. These findings highlight the need to align psychometric rigor with patient-centered values. A study is needed to evaluate the adequacy of existing QOL instruments, including Patient-Reported Outcomes Measurement Information System and existing QOL measures designed for older adults (WHOQOL-OLD, etc.) in a cohort of older dialysis patients, for both relevance and psychometric rigor. If no existing measures are a fit, then the development and validation of a QOL instrument for the older dialysis population would be the ideal next step. In addition, accepting patient-led approaches to identifying and addressing QOL priorities may be a pivotal next step in research in this area. Processes such as the measure yourself medical outcome profile^33^^,^^34^ and goal setting attainment^35^ may offer distinct approaches to focus on patient-identified goals and assess QOL interventions.

This study’s strength lies in the use of Q-methodology, a novel methodological approach that identifies perspectives by forcing a rank order of statements across the spectrum of opinions on a given topic. Factor analysis of these data leads to the creation of a unique perspective that is unlikely to be elicited from single-item Likert scale surveys. QOL priorities may be influenced by various factors, and instruments such as KDQOL-36 and WHOQOL-OLD are unable to capture the nuances related to varied patient preferences. Likert scale surveys are also subject to desirability and response-style bias and systematic error. Q-methodology circumvents the inherent limitations of Likert scales as participants are instructed to rank statements to establish a “hierarchy of agreements” and extract various viewpoints.^36^ Q- methodology also offers a valid and reliable approach for quantifying subjectivity, demonstrated by its high test-retest reliability.^14^ Further, large sample sizes are not required in Q-methodology because Q-methodology focuses on identifying shared viewpoints rather than estimating population-level effects.^37^ Despite the strengths of Q-methodology, our findings should be interpreted with the following limitations. First, the study was restricted to one geographic context in North Carolina, limiting its generalizability. Second, this study was cross-sectional by design and may represent viewpoints at the time of the study, and patient perspectives are subject to change over time while receiving dialysis.^22^ Last, the statements that were sorted (Q-set) were developed by the study team, which may have introduced researcher bias. Alternative approaches would include engaging participants in the process of Q-statement development.

## Conclusions

This Q-methodology study revealed 2 distinct QOL profiles, “Everyday Well-Being” and “Safety and Security.” This study represents a critical step toward more appropriate measurement of QOL in this population by highlighting the need for QOL assessments that cover a wide range of priorities. Such an approach may better support patient-centered care and intervention development.

## References

[1] United States Renal Data System (2024). https://adr.usrds.org/2024.

[2] De Pasquale C., Pistorio M.L., Corona D. (2012). Correlational study between psychic symptoms and quality of life among hemodialysis patients older than 55 years of age. Transplant Proc.

[3] Kallem C.J., Alghwiri A.A., Yabes J. (2024). Diurnal and daily symptom variation in patients with end stage kidney disease: an ecological momentary assessment study. Clin J Am Soc Nephrol.

[4] Devaraj S.M., Roumelioti M.E., Yabes J.G. (2023). Correlates of rates and treatment readiness for depressive symptoms, pain, and fatigue in hemodialysis patients: results from the TĀCcare study. Kidney360.

[5] de Rooij E.N.M., Meuleman Y., de Fijter J.W. (2022). Quality of life before and after the start of dialysis in older patients. Clin J Am Soc Nephrol.

[6] Hall R.K., Luciano A., Pieper C., Colón-Emeric C.S. (2018). Association of Kidney Disease Quality of Life (KDQOL-36) with mortality and hospitalization in older adults receiving hemodialysis. BMC Nephrol.

[7] Ishiwatari A., Yamamoto S., Fukuma S., Hasegawa T., Wakai S., Nangaku M. (2020). Changes in quality of life in older hemodialysis patients: a cohort study on dialysis outcomes and practice patterns. Am J Nephrol.

[8] van Oevelen M., Bonenkamp A.A., van Eck van der Sluijs A. (2024). Health-related quality of life and symptom burden in patients on haemodialysis. Nephrol Dial Transplant.

[9] Cohen D.E., Lee A., Sibbel S., Benner D., Brunelli S.M., Tentori F. (2019). Use of the KDQOL-36™ for assessment of health-related quality of life among dialysis patients in the United States. BMC Nephrol.

[10] Centers for Medicare & Medicaid Services (CMS), HHS (2008). Medicare and Medicaid programs; conditions for coverage for end-stage renal disease facilities. Final rule. Fed Regist.

[11] Butler C.R., Nalatwad A., Cheung K.L. (2025). Establishing research priorities in geriatric nephrology: a Delphi study of clinicians and researchers. Am J Kidney Dis.

[12] Hall R.K., Cary M.P., Washington T.R., Colón-Emeric C.S. (2020). Quality of life in older adults receiving hemodialysis: a qualitative study. Qual Life Res.

[13] Hays R.D., Kallich J.D., Mapes D.L., Coons S.J., Carter W.B. (1994). Development of the kidney disease quality of life (KDQOL) instrument. Qual Life Res.

[14] Brown S.R. (1993). A primer on Q methodology. Operant Subject.

[15] Cramm J.M., Leensvaart L., Berghout M., van Exel J. (2015). Exploring views on what is important for patient-centred care in end-stage renal disease using Q methodology. BMC Nephrol.

[16] Yeun E.J., Bang H.Y., Kim E.J., Jeon M., Jang E.S., Ham E. (2016). Attitudes toward stress and coping among primary caregivers of patients undergoing hemodialysis: a Q-methodology study. Hemodial Int.

[17] Lønning K., Midtvedt K., Heldal K., Andersen M.H. (2018). Older kidney transplantation candidates' expectations of improvement in life and health following kidney transplantation: semistructured interviews with enlisted dialysis patients aged 65 years and older. BMJ Open.

[18] Steinhauser K.E., Bosworth H.B., Clipp E.C. (2002). Initial assessment of a new instrument to measure quality of life at the end of life. J Palliat Med.

[19] Kelley N. (2009). An integrated conceptual model of quality of life for older adults based on a synthesis of the literature. Appl Res Qual Life.

[20] Morley J.E., Vellas B., van Kan G.A. (2013). Frailty consensus: a call to action. J Am Med Dir Assoc.

[21] Borson S., Scanlan J.M., Chen P., Ganguli M. (2003). The mini-cog as a screen for dementia: validation in a population-based sample. J Am Geriatr Soc.

[22] Elliott B.A., Gessert C.E., Larson P.M., Russ T.E. (2014). Shifting responses in quality of life: people living with dialysis. Qual Life Res.

[23] Liu C.K., Miao S., Giffuni J. (2023). Geriatric syndromes and health-related quality of life in older adults with chronic kidney disease. Kidney360.

[24] Crews D.C., Delaney A.M., Walker Taylor J.L. (2019). Pilot intervention addressing social support and functioning of low socioeconomic status older adults with ESRD: the Seniors Optimizing Community Integration to Advance Better Living with ESRD (SOCIABLE) study. Kidney Med.

[25] Kimmel P.L., Fwu C.W., Eggers P.W. (2013). Segregation, income disparities, and survival in hemodialysis patients. J Am Soc Nephrol.

[26] Crews D.C., Gutiérrez O.M., Fedewa S.A. (2014). Low income, community poverty and risk of end stage renal disease. BMC Nephrol.

[27] Ramer S.J., McCall N.N., Robinson-Cohen C. (2018). Health outcome priorities of older adults with advanced CKD and concordance with their nephrology providers' perceptions. J Am Soc Nephrol.

[28] Logan B., Ludlow K., Pascoe E.M. (2025). Goals of frail older people living with chronic kidney disease: a mixed methods study. J Am Geriatr Soc.

[29] Russwurm M., Rabaev A., Hoyer J.D., Haas C.S., Volberg C., Russ P. (2024). A survey on end-of-life contemplation among patients on dialysis. Kidney Int Rep.

[30] Song M.K., Manatunga A., Plantinga L. (2024). Effectiveness of an advance care planning intervention in adults receiving dialysis and their families: a cluster randomized clinical trial. JAMA Netw Open.

[31] Tsubaki M., Aoyagi H., Ito Y., Kobayashi M., Ushiwata A. (2025). Effects of advance care planning on the mental health of bereaved families: a systematic review. Cureus.

[32] Song M.K., Plantinga L., Metzger M. (2025). Implementation of an advance care planning intervention in dialysis clinics. Am J Kidney Dis.

[33] Lund-Tonnesen M., Thing Oggesen B., Vahr Lauridsen S., Fonnes S., Rosenberg J. (2025). The use of Measure Yourself Medical Outcome Profile (MYMOP): a scoping review. Dis Res.

[34] Lund-Tonnesen M., Oggesen B.T., Lauridsen S.V., Fonnes S., Rosenberg J. (2025). Good equivalence between electronic and paper versions of the measure yourself medical outcome profile 2 and the measure yourself concerns and wellbeing: a mixed methods study. Cureus.

[35] Turner-Stokes L. (2009). Goal attainment scaling (GAS) in rehabilitation: a practical guide. Clin Rehabil.

[36] Roberts J.K., Schub M., Singhal S. (2023). Exploring US internal medicine resident career preferences: a Q-methodology study. Adv Health Sci Educ Theory Pract.

[37] Roberts J.K., Hargett C.W., Nagler A., Jakoi E., Lehrich R.W. (2015). Exploring student preferences with a Q-sort: the development of an individualized renal physiology curriculum. Adv Physiol Educ.

