# Supplementary material for: Examining Quality-of-Life Priorities of Older Adults Receiving Hemodialysis: A Q-Methodology Study
Source: Kidney Med. 2026 Apr 1;8(6):101348. doi: 10.1016/j.xkme.2026.101348 (PMC13202277; doi:10.1016/j.xkme.2026.101348)
Supplement: Supplementary File (PDF) — Table S1. [file mmc1.pdf]

Supplementary Material

Table S1. Q-Statements on Quality of Life Priorities

**Statements**	
It is important that:	
1	I have support from family and friends.
2	I find strength in my faith or spiritual beliefs.
3	I feel safe in my home and neighborhood.
4	I am satisfied by the appearance of the dialysis unit.
5	I am treated with respect by dialysis nurses and doctors.
6	I keep my memory and thinking ability.
7	I have a dialysis access site that works without problems.
8	I have my pain controlled.
9	I have energy to do things I enjoy.
10	I don’t feel like a burden on my family and friends.
11	I am able to move around my home on my own.
12	I am able to take care of shopping, housework, managing money, and taking my medications on my own.
13	I am able to bathe, dress, eat, and go to the toilet on my own.
14	I can set my own schedule from day to day.
15	I don’t feel sick all the time.
16	I am satisfied with the care I receive from my healthcare team.
17	I have an active social life.
18	I do not feel lonely.
19	I am satisfied with how I have lived my life.
20	I am usually in good spirits.
21	I can help make decisions about my medical care.
22	I know what to expect as kidney failure affects my health.
23	I have restful sleep.
24	I feel I can help others.
25	I get along with my friends and family.
26	I recover quickly after each dialysis.
27	I have enough money to meet my needs.
28	I can talk to my loved ones about how I want to live my last days.
29	I have an active sex life.
30	I can travel when I want to.
31	I have someone to talk to about my problems.
32	I have someone to help with chores if I am not able to do them.

33	I have reliable transportation to and from dialysis.
34	I have things I look forward to doing.
35	I am not troubled by fear or worry much of the time.

a
